# The Effect of Community-Based Intervention on Dengue Awareness and Prevention Among Poor Urban Communities in Delhi, India

**DOI:** 10.34172/jrhs.2023.131

**Published:** 2023-12-29

**Authors:** Abhishek Lachyan, Rafdzah Ahmad Zaki, Bratati Banerjee, Nasrin Aghamohammadi

**Affiliations:** ^1^Center for Epidemiology and Evidence-Based Practice, Department of Social and Preventive Medicine, Faculty of Medicine, University of Malaya, 50603 Kuala Lumpur, Malaysia; ^2^Department of Community Medicine, Maulana Azad Medical College, New Delhi, India; ^3^School Design and the Built Environment, Curtin University Sustainability Policy Institute, Kent St, Bentley, 6102, Western Australia

**Keywords:** Community-based intervention, Dengue, Housing, Prevention

## Abstract

**Background:** This study aimed to assess the efficacy of a dengue intervention program in economically deprived urban regions of India, with a particular emphasis on housing conditions and community involvement. Given the global significance of dengue fever as a vector-borne disease, successful vector management requires effective community engagement.

**Study Design:** A quasi-experimental study.

**Methods:** This study was conducted with 314 participants from Delhi’s Sanjay Colony, divided into control and intervention groups. The study spanned 14 months (August 2020 to September 2021). The intervention program comprised two educational sessions held one month apart, covering dengue awareness, health self-care, and environmental maintenance. Data were collected at baseline, after each intervention session, and during a final follow-up assessment three months later.

**Results:** The primary outcome, the house index (HI), revealed statistically significant differences (*P*<0.001) favoring the intervention group. The total score (TS) for mosquito-borne disease, TS of knowledge, TS of attitude, and TS of practices all exhibited significant improvements in the intervention group. Participants showed an enhanced understanding of dengue causes, symptoms, and mosquito behavior related to breeding and biting. The HI in the intervention group decreased significantly from 21.65% to 4.45% (*P*<0.05).

**Conclusion:** This study, grounded in the health belief model (HBM), demonstrated the effectiveness of the intervention program in reducing HI and improving knowledge and preventive practices regarding dengue fever in impoverished urban neighborhoods of Delhi. The intervention program may be beneficial in such a poor urban community.

## Background

 Dengue fever, a rapidly spreading virus transmitted by mosquitoes, stands as a formidable global public health challenge.^1^ The magnitude of this problem is underscored by the staggering surge in cases by a factor of 30 over the past five decades. According to the World Health Organization, dengue has witnessed a worldwide incidence rate increase of approximately 30% annually. The mortality rate, though comparatively lower, is significant, with approximately 20 000 deaths reported globally each year.^2,3^ Additionally, the disability caused by dengue, including the long-term health impacts on survivors, adds another layer of concern, affecting approximately 500 000 individuals annually.^4^

 In India, where the climate is conducive to dengue transmission, the National Vector Borne Diseases Control Programme (NVBDCP) has been actively engaged in prevention and management efforts.^5^ However, the incidence rate of dengue in the country remains a critical concern, particularly after the monsoon season, when Aedes mosquitoes, the primary vectors, thrive in artificial water containers near homes, factories, and construction sites.^6^

 Effective dengue control demands a comprehensive strategy, encompassing vector surveillance, integrated mosquito management, environmental upkeep, legislation, and crucially, community involvement.^7^ Health education emerges as a linchpin in this strategy, and the NVBDCP employs diverse communication channels to educate populations in endemic regions.^8^

 The health belief model (HBM), rooted in social psychology theory, informs the preventive strategies of this study. According to the HBM, individuals are more likely to adopt disease prevention practices when they perceive the benefits, encounter fewer barriers, receive cues to prompt action, face modifying factors, and possess the motivation to stay healthy.^9-12^ Recognizing the pivotal role of community involvement in dengue control, this study focuses on raising awareness, enhancing the understanding of disease risks, emphasizing prevention benefits, and removing obstacles to preventive actions.^13-18^ The goal is to assess how a community-based intervention can contribute to increased awareness and reduced dengue incidence, particularly in the impoverished urban areas of Delhi.

## Methods

 The purpose of this study is to examine the effectiveness of the intervention program for dengue fever prevention among poor urban communities in Delhi, India, based on the theory of HBM.

###  Study Design and Duration

 As mentioned earlier, this quasi-experimental research aimed to evaluate the effectiveness of dengue fever prevention strategies in poor urban communities. With a control group, this study employed a quasi-experimental approach. Following the session, this group completed a post-intervention evaluation. A control group receives pre- and post-tests and no intervention simultaneously as the other groups. These research techniques are often used to investigate educational programs’ effectiveness.

**Figure 1:**
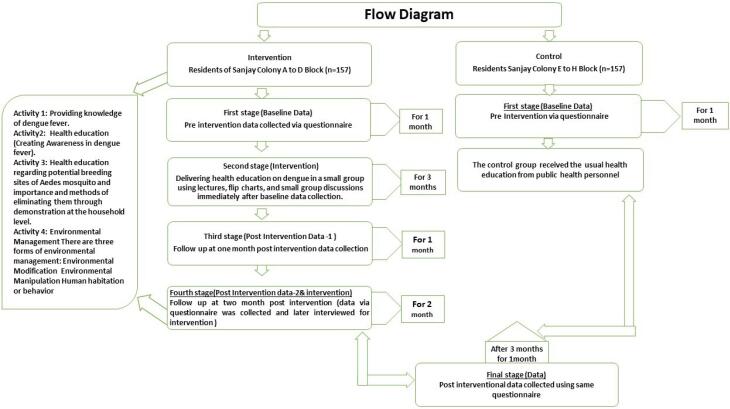
The Study Flow Chart

**Figure 2:**
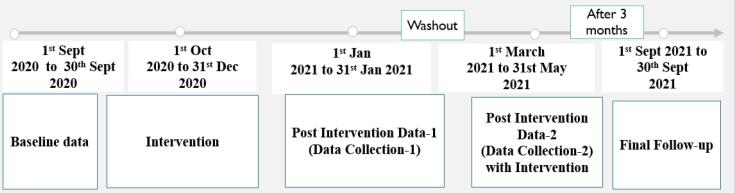
Timeline of Research

 Studies to evaluate interventions without the use of randomization are called quasi-experiments. Similar to randomized trials, quasi-experiments seek to show that an intervention causes an effect. Preintervention and postintervention assessments and groups that are not randomly selected can all be utilized in quasi-experimental studies. This study could not adopt a randomized control trial design because participant randomization took a lot of work. Randomizing the intervention for each participant is frequently tricky. The other reason for not adopting the mentioned design was the difficulty of location-based randomization (e.g., colony). Similar difficulties arise when attempting to randomize interventions to specific places, as well as dengue therapies to specific sites. Using the dengue knowledge, attitude, and practice (KAP) example, applying the system in some hospitals or areas might be challenging. Let us declare that the dengue KAP includes a learning component. If that happened, it may appear that the intervention was unsuccessful because people would likely apply what they had learned to a colony that was not a member of the KAP. Participants were randomly divided into a control group continuing their routine practices and an experimental group undergoing a three-month intervention (Figure 1). Data were collected before (baseline), immediately after, and during a final follow-up (3-month intervention and 1-month final follow-up). The study was conducted between August 2020 and September 2021 (Figure 2).

###  Participant Selection

 Residents aged 18 years were included, and one member was randomly selected in households with multiple eligible members. The inclusion criteria also encompassed a residency of over a year in the study area, the ability for effective communication, and the willingness to participate. On the other hand, the exclusion criteria were applied to individuals with serious illnesses and conditions affecting participation.

###  Sampling Method

 The study utilized systematic random sampling to select households, applying a formula to calculate the sampling interval (k) and ensuring a sample size of 157 for each group. The sample size needed for this study was 157 for each group.

###  Questionnaire Structure

 The questionnaire structure employed in the study followed a pre-tested, closed-ended format. To ensure the validity and reliability of the questionnaire, content validity, including expert validation of the questionnaire and measurement instrument, was assessed through testing. Additionally, the reliability of the questionnaire was confirmed using Cronbach’s alpha coefficient.

###  Interventions Implemented

 In the study, participants in the control group received no interventions, serving as a comparison against those in the intervention group. Participants in the intervention group were enrolled in a program guided by the HBM theory. The interventions implemented in this group were diverse, incorporating a range of strategies aimed at bolstering community awareness and fostering engagement in dengue prevention efforts. Further details about these interventions are provided in Table 1 and Figure 1.

**Table 1:** The details of interventions implemented in the study

**Activity**	**Description**
Public awareness on dengue fever	Daily local broadcasts designed based on the health belief model components: Increasing susceptibility, highlighting severity, emphasizing benefits, and reducing barriers
Health education (Creating dengue fever awareness)	- Dissemination of health information through lectures, flip charts, and small group discussions - Distribution of pamphlets and posters on dengue prevention techniques and guidelines from the Dengue Control Division - Information on using Temephos (Abate sand) and insect repellent to eliminate larvae - Distribution of pamphlets with information about dengue disease, including warning signs and symptoms
Household-level health education on Aedes mosquito breeding	Face-to-face training sessions with families, covering Aedes mosquitoes’ breeding sites and emphasizing prompt cleaning and container covering Community-level education included street plays to raise awareness The standardized larval survey format was conducted in 157 homes, calculating house index, container index, and Breteau index
Environmental management	Collaboration with community leaders and Delhi’s Municipal Corporations. Environmental modification, manipulation, and human habitation or behaviour approaches implemented to prevent dengue Municipality incorporated recommendations post-study, resulting in infrastructure improvements and enhanced essential services

###  Data Collection Instrument

 The questionnaire was structured into sections on socio-demographics, knowledge, attitude, and preventive practices. The questions were designed to assess participants’ understanding, attitudes, and behaviors related to dengue fever.

*Section I: Socio-demographic profiles:* This section aimed to gather essential socio-demographic information about the participants, providing a foundation for understanding the context in which their KAPs regarding dengue fever were situated.

*Section II: Knowledge:* Comprising 21 questions, this section focused on assessing participants’ understanding of the risk factors associated with dengue fever. Each question was scored with 1 for ‘Yes’ and 0 for ‘No’, and the overall knowledge was evaluated using mean scores, offering a quantitative measure of participants’ comprehension of dengue fever.

*Section III: Attitudes:* This section, consisting of 6 questions, delved into participants’ attitudes toward dengue fever as a disease. Responses were measured on a Likert-type scale, ranging from 5 (Strongly agree) to 1 (Not sure). Mean scores were again used to gauge the overall attitude of the study participants, providing insight into their conscious awareness of dengue fever risk factors.

*Section IV: Preventive Practices:* With 19 questions, this section focused on participants’ self-reported practices to prevent dengue fever. The responses were scored as 1 for ‘Yes’ and 0 for ‘No’, reflecting the implementation of preventive measures. This section sought to capture the practical aspects of participants’ behavior related to dengue fever prevention.

 The study applied a systematic approach by incorporating a combination of closed-ended questions, Likert-type scale responses, and scoring mechanisms to quantitatively evaluate the KAP of the target population regarding dengue fever. The use of mean scores facilitated a more nuanced understanding of the participants’ overall awareness, attitudes, and behavioral practices in the context of dengue fever, contributing valuable insights into public health interventions and education campaigns.

 To ensure dependability, 30 individuals possessing similar characteristics as the study samples were employed to evaluate the questionnaire’s reliability. The internal consistency and reliability of the questionnaire were confirmed through the examination of Cronbach’s alpha (α) coefficient, which yielded excellent internal consistency values of at least 0.80 across all scales. The clarity, informativeness, and overall coherence of the questions underwent assessment. All questions were formulated as open-ended queries, and the responses were meticulously reviewed and grouped based on similarities. Data entry and descriptive analysis were conducted using SPSS software, version 25.

###  Intervention Program Evaluation

 To assess the effectiveness of the intervention program, data were collected before, immediately after, and during a final follow-up. Changes in the house index (HI) were a primary outcome measure, demonstrating a significant decrease in the intervention group.

###  In-Depth Analysis

 The study aimed to facilitate in-depth analysis by employing a quasi-experimental design. This design allowed for a comprehensive evaluation of the intervention’s impact in a real-world setting, considering the complexities of poor urban communities in Delhi. The quasi-experimental approach was chosen due to the practical challenges of participant randomization and location-based randomization. These challenges, including the intricate process of randomizing interventions to specific places, were addressed by adopting a quasi-experimental design, ensuring a pragmatic yet rigorous evaluation of dengue prevention strategies.

###  Statistical Analysis

 The information was gathered on a Microsoft Redmond Campus MS Office Excel Sheet (version 2019), Redmond, Washington, USA. The data were examined using SPSS (version 25.0, IBM), a statistical package for the social sciences. A coding system that has been checked for entry errors is provided by statistical analysis to reveal all the data entered into the computer. Descriptive statistics, such as frequencies and percentages, as well as means and standard deviations (SD), have been shown for categorical and numerical data, respectively. The chi-square test compared the frequencies of various variable categories within the group. A t-test was used to compare the two groups, and a repeated measures ANOVA (for more than two observations) and a post hoc test were employed for comparison within the same group. With an error of between 5% and 20% for all statistical tests, *P* > 0.05 was deemed statistically significant, giving the study power of 80%. The following notations apply to all tables:

^#^ = Non-significant difference (*P*> 0.05), ^**^ = Statistically very significant difference (*P*< 0.01), and ^*^ = Statistically significant difference (*P*< 0.05).

 The difference in the number of HIs between the two groups was analyzed using the χ^2^ test.

## Results

Table 2 presents a comprehensive breakdown of the distribution and comparison of demographic factors between the control and intervention groups. The factors influencing the desired outcomes do not differ between the control and intervention groups; the baseline sociodemographic characteristics of both groups were the same (Table 1). Apart from occupation, where it was observed that the majority of participants were employed in various industrial settings, there were no significant variations in the overall characteristics of the intervention and control groups.

**Table 2:** Comparison of thesociodemographic differences between the intervention and control groups

**Variables**	**Control (n=157)**		**Intervention (n=157)**		**χ**^2^	***P***** value**
	**Number**	**Percent**	**Number**	**Percent**		
Gender						
Male	78	49.69	77	49.04	0.000	1.000
Female	79	50.31	80	50.96		
Age						
18-27	40	25.47	38	24.20	6.684	0.154
28-37	44	28.02	37	23.56		
38-47	46	29.29	40	25.47		
48- 57	21	13.37	25	15.95		
58 and above	6	03.85	17	10.82		
Education						
Illiterate	14	08.91	4	02.56	10.482	0.063
Primary	72	45.88	73	46.49		
Secondary	41	26.11	58	36.94		
Collegiate	30	19.10	22	14.01		
Occupation						
Unemployed	18	11.46	7	04.45	54.024	0.001
Housewife	45	28.66	23	14.64		
Student	3	01.94	6	03.82		
Aged population	6	03.82	10	06.36		
Private job	46	29.29	25	15.96		
Government job	8	05.09	00	00.00		
Self-employed	31	19.74	86	54.77		
Types of family						
Nuclear family	131	83.43	135	85.99	0.393	0.530
Joint family	26	16.57	22	14.01		
Religion						
Hindu	153	97.45	153	97.45	0.140	0.709
Muslim	4	02.54	4	02.54		
Marital status						
Married	150	95.46	145	92.35	1.401	0.237
Unmarried	7	04.45	12	07.65		
Income (Rs)						
≥ 7533	157	100	151	96.17	6.117	0.047
3766-7532	0	00.00	5	03.18		
1130-2259	0	00.00	1	0.65		

Table 3 provides an initial comparison of total scores (TS) between the intervention and control groups, emphasizing factors such as mosquito-induced illnesses, knowledge, attitude, and behaviors. The initial “t” value for the variable “Disease caused by mosquitoes” was -0.830. However, upon closer examination, no statistically significant difference between the groups was observed at the beginning (*P*-value > 0.05).

**Table 3:** Intergroups comparison of scores at baseline total score (n = 157)

**Variables**	**Mean**	**SD**	**SE**	***P***** value**
Total score for disease caused by mosquitoes				
Control	1.92	0.967	0.077	0.407
Intervention	2.01	0.937	0.075	
Total score knowledge				
Control	10.48	3.407	0.380	0.788
Intervention	10.64	2.871	0.453	
Total score attitude				
Control	20.71	4.497	0.359	0.777
Intervention	20.83	3.398	0.271	
Total score practices				
Control	4.37	1.882	0.150	0.077
Intervention	3.99	1.869	0.149	

*Note*. SD: Standard deviation; SE: Standard error.

 Based on the data (Table 4) obtained in the final follow-up, the “t” value for the variable “Disease caused by mosquitoes” was 30.316 when comparing the intervention and control groups. Subsequent analysis indicated a statistically significant difference between the intervention and control groups concerning the variable “Disease caused by mosquitoes” (*P* value = 0.001).

**Table 4:** Intergroups comparison of scores at final (3-month) follow-up (n = 157)

**Variables**	**Mean**	**SD**	**SE**	***P***** value**
Total score for disease caused by mosquitoes				
Intervention	5.68	0.470	0.037	0.001
Control	3.27	0.874	0.070	
Total score of knowledge				
Intervention	25.55	0.720	0.057	0.001
Control	12.81	3.193	0.255	
Total score of attitudes				
Intervention	21.06	2.480	0.198	0.001
Control	18.24	5.610	0.448	
Total score of practices				
Intervention	16.00	0.000	0.000	0.001
Control	4.37	1.882	0.150	

*Note*. SD: Standard deviation; SE: Standard error.

Table 5 provides a summary of larval index measurements before and after the intervention, indicating a reduction in larvae in breeding sites. Initially, there were multiple baseline intervention instances at the locations (Larvae = 34, HI = 21.65) and baseline control group instances (Larvae = 31, HI = 19.74). However, after the intervention, there was a noticeable decrease in dengue cases in the intervention block compared to the control group (Larvae = 29, HI = 18.47).

**Table 5:** Comparison of the number of dengue cases, larvae, and HI between intervention and control groups

**Phase**	**Intervention Group (n=157)**		**Control Group (n=157)**		**χ**^2^	***P*****-value**
**Measuring Time**	**Found Larvae (n)**	**HI**	**Found Larvae (n)**	**HI**		
Intervention baseline	34	21.65	31	19.74	0.417	0.674
Post-intervention data-1	7	04.45	-	-	2.675	0.007
Post-intervention data-2	0	0	-	-	-	-
Final follow-up	0	0	0	29	5.656	0.001

*Note*. HI: House index.

## Discussion

 In the discussion, percentages and detailed results have been omitted, focusing on the interpretation of positive and negative outcomes, explanations, and references to relevant studies.

 The intervention program aimed at enhancing dengue awareness and prevention within a poor urban community in Delhi, India, demonstrated significant positive outcomes. The study applied the HBM as the theoretical framework, focusing on knowledge, perceived susceptibility, perceived severity, perceived benefits, perceived barriers, and preventive action.^19^

 The intervention program aligned with the HBM theory, recognizing the importance of knowledge, perceived susceptibility, perceived severity, perceived benefits, and perceived barriers in shaping protective behaviors. The program was designed to bridge the gap between awareness and action, addressing the challenge of translating knowledge into practical measures to reduce mosquito populations.^20^

 Various strategies were employed to enhance knowledge, including daily broadcasts, community-wide dengue campaigns, and group education sessions at the village hall. Additionally, a contest for a model house for dengue safety served as a cue to action, encouraging participants through positive community modeling. This multi-faceted approach aimed to foster a comprehensive understanding of dengue and promote preventive actions.^21^

 Post-intervention, participants in the intervention group exhibited significantly higher scores across the six scales related to dengue hemorrhagic fever prevention compared to the control group. This outcome underscores the direct effectiveness of the intervention program. However, during the follow-up phase, some scores decreased, emphasizing the need for sustained community engagement to maintain the positive effects.^22^

 The study highlighted the importance of community involvement in sustaining preventive actions. The decrease in scores during the follow-up phase suggests that continued efforts to enhance community engagement are crucial for long-term success. Encouraging behavioral change for dengue hemorrhagic fever prevention requires ongoing support, emphasizing the need for a sustained and collaborative approach within the community.^23^

 The study observed a decrease in the HI for the intervention group post-intervention and during the follow-up. This decrease indicates the positive impact of the program on preventive actions, aligning with previous research linking knowledge of preventive measures to practical practices. To effectively reduce mosquito breeding sites, a community-wide shift in behaviors and sustained efforts are necessary.^24-26^

 The study acknowledges potential reporting and social desirability bias, partly attributed to the researchers’ involvement. While structured survey techniques mitigate investigator influence, participant observation limitations were taken into consideration. Despite these drawbacks, the study emphasizes the value of health education initiatives in raising awareness and changing practices. Random sampling enhances the generalizability of findings.

**Highlights**

- Tailored communication to address specific challenges faced by the urban poor
- Engaged communities through workshops, awareness campaigns, and forums
- Implemented innovative educational tools for effective information dissemination
- Measurable improvements in dengue awareness levels and the adoption of preventive measures
- Enhanced community collaboration with healthcare providers
- Findings inform public health policies for urban areas
- Recommendations for scaling similar interventions in resource-constrained settings

## Conclusion

 In general, the findings of this present study underscored the effectiveness of community-based interventions in enhancing knowledge and fostering the adoption of specific protective behaviors within impoverished urban areas. It is noteworthy that the focus of the intervention was not on measuring physical structures but on educating and engaging the local population.

 While the study findings demonstrated that educational efforts can lead to positive behavioral changes, they also emphasized the insufficiency of education alone. Robust vector control measures and community-based environmental management strategies are essential complements to educational initiatives. These additional measures are crucial for inducing lasting behavior changes and, ultimately, eliminating diseases such as dengue.

 Moreover, the study highlights the importance of providing healthcare workers with enhanced training in community engagement and communication. This aspect is imperative, especially in impoverished urban areas with a high prevalence of dengue fever. The HBM-based dengue fever prevention initiative employed in this study has proven successful in reducing the HI and increasing knowledge, perception of vulnerability, understanding of severity, overcoming barriers, and the adoption of preventive actions among residents in underdeveloped urban areas.

 Therefore, the intervention program emerges as highly beneficial in effectively addressing the dengue issue within such an underprivileged metropolitan population. The findings suggest that a multifaceted approach, combining education, vector control, and community engagement, is crucial for sustained success in mitigating vector-borne diseases in similar contexts.

## Acknowledgments

 I express deep gratitude to Almighty God for His blessings throughout my journey. The success of this project is indebted to those who offered invaluable support, guidance, and feedback. I extend my sincere thanks to my supervisor, Associate Professor Dr. Rafdzah Binti Ahmad Zaki, for her patience and unwavering support during the field study. I also acknowledge the commitment of Associate Professor Dr. Nasrin Aghamohammadi and Professor Dr. Bratati Banerjee for their invaluable contributions. Special appreciation goes to the Department of Social and Preventive Medicine at the University of Malaya, my family for their love, and friends for their unwavering support. I am eternally grateful for the collaborative efforts that brought this project to fruition.

## Authors’ Contribution

**Conceptualization:** Abhishek Lachyan, Rafdzah Ahmad Zaki.

**Data curation:** Abhishek Lachyan.

**Formal analysis: **Abhishek Lachyan, Rafdzah Ahmad Zaki.

**Investigation:** Abhishek Lachyan, Bratati Banerjee.

**Methodology:** Abhishek Lachyan, Rafdzah Ahmad Zaki, Bratati Banerjee, Nasrin Aghamohammadi.

**Project administration:** Abhishek Lachyan.

**Resources:** Abhishek Lachyan, Rafdzah Ahmad Zaki, Bratati Banerjee, Nasrin Aghamohammadi.

**Software:** Abhishek Lachyan.

**Supervision:** Bratati Banerjee, Rafdzah Ahmad Zaki, Nasrin Aghamohammadi.

**Validation:** Bratati Banerjee.

**Visualization:** Bratati Banerjee.

**Writing–original draft:** Abhishek Lachyan.

**Writing–review & editing:** Abhishek Lachyan, Rafdzah Ahmad Zaki, Bratati Banerjee, Nasrin Aghamohammadi.

## Competing Interests

 None declared.

## Ethical Approval

 The study received ethical approval from the University of Malaya Research Ethics Committee (UMREC) Malaysia (reference number UM.TNC 2/UMREC dated April 5, 2020) and the Institutional Ethics Committee (IEC) Maulana Azad Medical College and Associated Hospital, New Delhi, India (F.1/IEC/MAMC/79/07/2020/No.204 Dated on August 26, 2020).

## Funding

 There are no funding sources.
